# Dose-dependent effect of *N*'-Nitrosodiethylamine on hepatic architecture, RBC rheology and polypeptide repertoire in Wistar rats

**DOI:** 10.1515/intox-2015-0001

**Published:** 2015-03

**Authors:** Devoshree Mukherjee, Riaz Ahmad

**Affiliations:** Biochemical and Clinical Genetics Lab, Section of Genetics, Department of Zoology, Aligarh Muslim University, Aligarh-202002 (U.P), India

**Keywords:** hepatotoxicity, *N*'-Nitrosodiethylamine, oxidative stress, polypeptide repertoire, RBC, SDS-PAGE

## Abstract

*N*'-Nitrosodiethylamine (NDEA) is an effective hepatotoxicant, carcinogen and mutagen. NDEA-induced hepatic necrosis, through metabolic activation by CYP2E1, is an extensively used experimental model. In the present study, we analysed the dose- and time-dependent effect of NDEA on hepatic damage, RBC rheology and proteomic profile in male Wistar rats. The rats, 5–6 weeks old, were divided into four groups: Group-1 served as control and received normal saline, Group-2 received a single dose of 200 mg/kg body weight NDEA intraperitoneally (i.p.) and the animals were sacrificed after one week; the rats of Group-3 received a single dose of 100 mg/kg body weight NDEA and were sacrificed after one week; Group-4 received 100 mg/kg body weight/wk NDEA for two weeks and were then sacrificed. Various biochemical parameters such as ALT, AST, ALP and bilirubin were determined. Further, RBC rheology, histopathology (H&E staining) of liver biopsies and polypeptide profiling (SDS-PAGE) in sera and liver sections were also carried out both in control and NDEA treated groups. Our results showed a significant increase in all the biochemical parameters of the liver function test (*p*<0.05). In NDEA treated categories dacryocytes (tear drop cells), schistocytes (fragmented cells), codocytes (target cells), acanthocytes (spur cells) and ovalocytes (oval cells) were observed. H & E stained liver biopsies treated with NDEA showed abnormal liver architecture with severe haemorrhage, neutrophilic infiltration and dysplastic hepatocytes manifested in a dose-dependent manner. Software analysis of SDS-PAGE of control and NDEA treated rat sera and liver revealed qualitative and quantitative differences in polypeptide composition. Based on the presence/absence, polypeptides were classified in three different categories: (1) house-keeping, present in all the groups investigated; (2) novel, present in either control or NDEA treated group at any given time; (3) differential expression, showing quantitative differences. Our study indicates a dose and time-dependent hepatocellular damage and proteome profile which is likely due to NDEA-mediated oxidative stress in rats.

## Introduction

Nitrosamines are known to be an important class of carcinogens posing a significant threat to human health (Abut *et al*., 2003). The most commonly occurring nitroso compounds are *N*'-Nitrodimethylamine (NDMA), Nitrosopyrrolidine, Nitropiperidine and *N*'-Nitrosodiethylamine (NDEA) (Brambilla *et al*., 1987). The presence of many of them has been detected in food, drugs, beverages and tobacco throughout the world (Choi *et al*., 2002; Xia *et al*., 2005). NDEA is one of the potent carcinogenic dialkyl nitrosamine agents that are identified in smoke and waste-water, in addition to the above-mentioned sources (Kumar & Kuttan, 2000). Besides endogenous synthesis of NDEA in an empty stomach on taking pesticide-rich food, this compound has been recorded in elevated concentrations ranging from 4.8 μg/kg in corn bread to 10–20 μg/kg in Chinese seafood, sausages and cheese (Lijinsky, 1999). In this context, investigations on the possible effect of these compounds may be of global interest. In the present study, we selected NDEA to investigate the damage caused by this nitroso compound in a mammalian model *in vivo.*

The liver, the largest internal glandular organ, is structurally and functionally heterogeneous in vertebrates. It performs many important functions such as detoxification, fat digestion, nutrient processing, urea and cholesterol production, vitamin, glycogen and mineral storage, along with blood infiltration. There are, however, many factors responsible for causing altered functioning of the liver leading to various diseases. At present, liver damage is most frequently caused by food additives, alcohol, toxic industrial chemicals, viral infection, air and water pollutants (Farazi *et al*., 2006; Jemal *et al*., 2007; Ahmad *et al*., 2012a). Hepatotoxicity always occurs by impaired hepatocyte metabolism and sometimes by distinctive deposition of connective tissue components in the liver (Matsui *et al*., 1994; George, 2006).

In humans, endogenous synthesis of nitrosamines is the consequence of the reaction between the nitrite ion and secondary/tertiary amines at low pH (Lijinsky, 1999; Oshawa *et al*., 2003). Basically, nitrites are formed by the reduction of nitrates. These nitrates are used to preserve various foods (especially meat products) and to develop flavour and colour. Since the liver is the primary site of drug metabolism, it is intriguing to know the effect of nitrosamines on liver function. To our understanding, any liver injury induced by hepatotoxins like D-Galactosamine, CCl_4_, NDMA, NDEA or alcohol primarily stimulates the Kupffer cells to secrete various cytokines (such as IL-1, 6 and 8) and chemokines (like MIP-2, IP-102, MIP-1α, MCP-1). These secreted molecules cause liver injury either directly or indirectly through chemo-attraction of neutrophils and lymphocytes (Ahmed & Vernick, 2011). In this study, we administered different doses of NDEA to Wistar rats and monitored the changes in a dose- and time-dependent manner. To assess the detrimental effects of NDEA on experimental animals, liver function test (LFT) markers, RBC rheology, histopathology (H&E staining) of liver biopsies and polypeptide profiling under denatured conditions were taken as parameters.

## Materials and Methods

### Chemicals and reagents

Acrylamide, Bis-acrylamide, Tris, Ammonium per sulphate (APS), *N,N,N',N'*-Tetramethylethylenediamine (TEMED) and *N*'-Nitrosodiethylamine (NDEA) were purchased from Sigma-Aldrich. Coommassie Brilliant Blue (CBBR-250), Haematoxylin and Eosin Stains along with Bovine Serum Albumin (BSA) were obtained from SRL. All other chemicals and reagents used were of AR grade.

### Care and maintenance of animals

A total of twenty healthy male Wistar rats, *Rattus norvegicus,* five to six weeks old, (150±10 g) were selected for this study. The animals were housed in well aerated polycarbonate cages with a bed of husk under hygienic conditions with proper humane care at the animal house facility in the department. The animals were maintained at light: dark exposure of 12:12 h at constant RT (~28±2 °C) with controlled humidity and proper air circulation. Prior to experimentation, the animals were acclimatised for about a week under laboratory conditions and fed regularly with commercially available sterilised diet (Ashirwad Industries Pvt. Ltd., Mohali, Punjab, India) and water available *ad libitum.* All the experiments were carried out according to the guidelines of the Committee for the Purpose of Control and Supervision of Experiments on Animals (CPCSEA), New Delhi, India.

### Administration of *N*'-Nitrosodiethylamine (NDEA) to induce hepatic damage

To induce liver damage, NDEA injections were prepared by suspending it in sterilised saline. The injections were given to individual groups comprising five rats each, as per the following protocol.

Group-1: The animals received 1 ml of normal saline only. This group served as control.

Group-2: The animals received a single intraperitoneal (i.p.) injection of NDEA of 200 mg/kg body weight and were sacrificed after a week.

Group-3: The animals received a single i.p injection of NDEA of 100 mg/kg body weight and were sacrificed after a week.

Group-4: The animals received i.p injections of NDEA of 100 mg/kg body weight per week for two weeks and were sacrificed for further investigations.

### Collection of blood and sera

To collect blood through cardiac puncture, the animals were anaesthetised. Approximately 1.5–2 ml blood was collected from each rat of either group. Smears were prepared on clean and sterilised slides using about 1–2 drops of blood, while the remaining blood was kept at room temperature to ooze out the sera (Ahmad *et al*., 2014a). Slides were fixed in methanol and subsequently stained with Giemsa to study RBC structure. The pale yellow coloured serum was collected by low-speed centrifugation and analysed afresh, or otherwise it was stored in aliquots at –20 °C for further analysis.

### Histological assessment of liver damage

The degree of hepatic damage due to NDEA treatment was examined by haematoxylin and eosin (H&E) staining of serial liver sections following the standard protocol (Ahmad *et al*., 2009). Stained slides were examined and the best fields were photographed under Microscope (Olympus BX-50) equipped with an LCD attachment.

### Preparation of liver homogenates

Following total body and liver weight assessment, the excised livers of the sacrificed animals were homogenised in chilled 50 mM Tris-HCl buffer, pH 7.5 (Ahmad *et al*., 2014b). The homogenates were centrifuged at 8,000 rpm for 20 min. The clear supernatant was collected and stored at –20 °C in different aliquots for further analysis.

### Estimation of biochemical parameters and assessment of RBC rheology

Routine sera biomarkers of liver function test (LFT) such as glutamic oxaloacetic transaminase (GOT/AST), glutamic pyruvate transaminase (GPT/ALT), alkaline phosphatase (ALP) and total bilirubin were determined using commercial kits obtained from Erba, Coral and AutoZyme Diagnostic Limited. To assess alterations in RBC shape as a result of NDEA toxicity, blood smears were examined under microscope (Motic: BA-210) and photographed at appropriate magnifications.

### Protein determination

Protein concentration in the sera and liver homogenates were determined at room temperature following the protocol of Lowry *et al*. (1951) and taking bovine serum albumin as standard. Colour in the solution was read at 660 nm on UV-VIS spectrophotometer (Bio Sync, India).

### Molecular weight-based polypeptide profiling of sera and liver homogenates

SDS-polyacrylamide gel electrophoresis (SDS-PAGE) of sera and liver samples was carried out in 10% vertical slab gel (80×100×1 mm) according to the protocol outlined by Laemmli (1970). Equal amounts of proteins were loaded and the sample was allowed to enter the resolving gel slowly and later the gels were run at 20 mA, 110 V/ gel till completion. The gels were subsequently washed overnight in 7% acetic acid at 37 °C and stained in CBBR-250 for 10–15 mins. Destaining was carried out in 5% acetic acid. The best gels were either scanned or photographed under proper illumination. Molecular weight of polypeptides was estimated by using freshly prepared chicken actomyosin as a molecular weight marker.

### Densitometry and quantitative assessment of PAGE profiles

The quantitative evaluation of polypeptide profiles were made by densitometry of the gel-scans using Scion Imaging (Scion Corporation: Beta release-4.0) and GelPro (Media Cybernetics, USA) software programmes.

### Statistical analysis

Student's t-test was applied to find the differences between the treatment groups and control. The values were considered statistically significant at *p*<0.05.

## Results

During the present study, we carried out toxicological evaluation of *N*'-Nitrosodiethylamine (NDEA) in male albino rats using some biochemical, cytological and histological parameters. Our results showed that NDEA induced hepatocellular damage in a dose- and time-dependent manner. The damage was clearly evidenced by the marked elevation in the levels of serum ALT, AST and ALP, along with altered RBC rheology and polypeptide profiling. The supportive data, obtained by histopathology, were also in favour of the above findings.

### Animal body and liver weight

**Figure 1:**
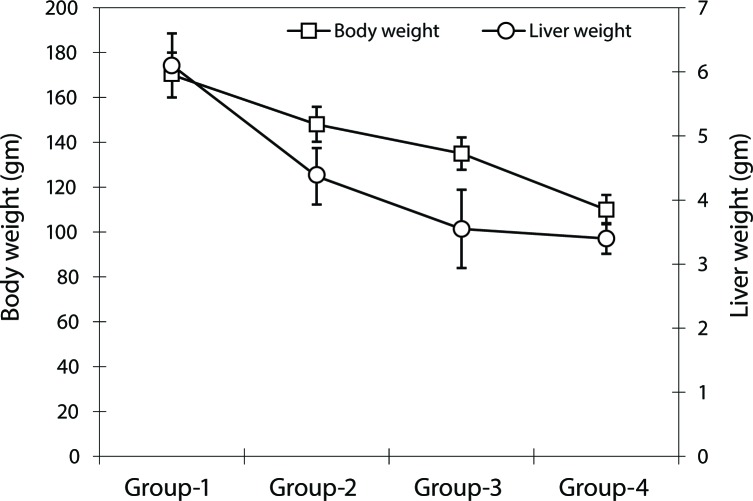
Changes in the body and liver weight of control and NDEA-treated rats during progression of hepatic damage.

A significant decline in body and liver weight of the rats was observed in the NDEA-treated groups compared to the control (*p*<0.05; Figure 1). The recorded difference in liver weight of the animals belonging to Groups-3 and -4 was however non-significant. The decrease in body and liver weight of the animals followed a time-dependent course.

### Changes in sera GOT, GPT, ALP and bilirubin

**Figure 2:**
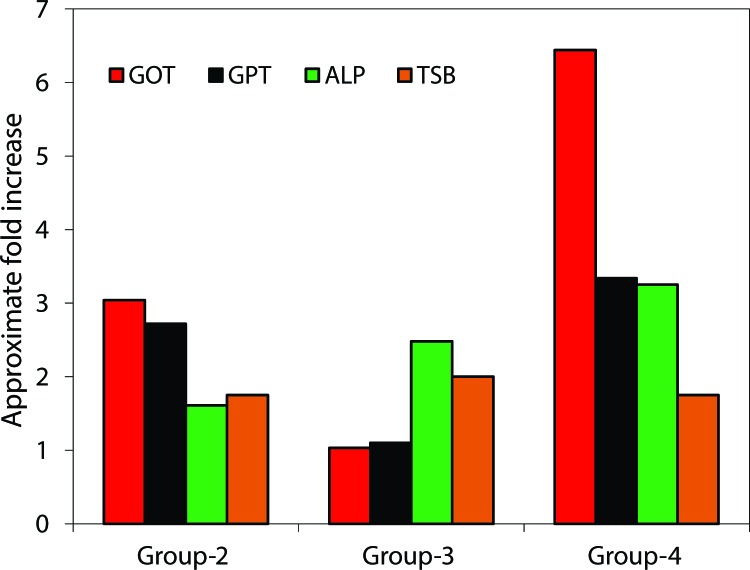
Bar-diagrams showing fold change in the liver function test biomarkers in control and NDEA treated rats.

Hepatic damage due to NDEA in rats was assessed by the levels of biochemical markers of liver function such as serum GOT, GPT, ALP and bilirubin. The obtained levels of these parameters are given in Figure 2. As a result of NDEA administration, a marked elevation in the biomarkers investigated was observed in the sera of rats at every selected dose. In Group-3, the magnitude of increase in the investigated biomarkers was the lowest compared with the other groups. The highest increase in the activity of the above biomarkers was observed in animals belonging to Group-4, *i.e.* those that received NDEA in 100 mg/kg body weight/wk for two weeks.

### RBCs rheology

**Figure 3:**
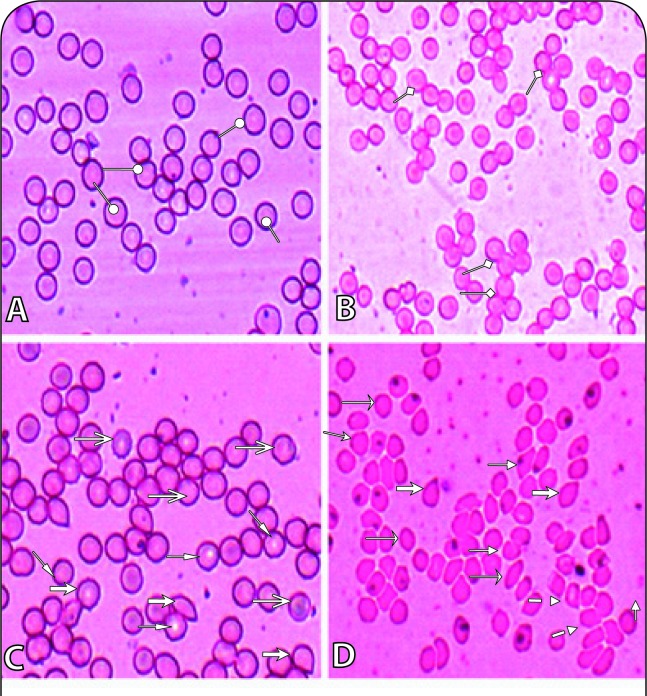
Plates showing RBCs stained with Giemsa. (A) Group-1 (Control): Normal RBCs without any alterations were observed. (B–D: Group-2–4): RBCs with different shapes were detected and photographed. 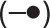 = Normal; 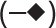 = Agglutination; 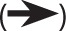 Tear drop cells; 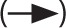 = Limon cells; 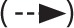 = Fragmented cells; 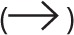 = Ovalocytes; 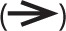 = Acanthocytes; 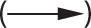 = Target cells.

Observations on peripheral blood smears indicated altered phenotypes of RBCs due to NDEA toxicity. Different types of cell shapes such as dacryocytes (tear drop cells), schistocytes (fragmented cells), codocytes (target cells), acanthocytes (spur cells) and ovalocytes (oval cells) were present in NDEA treated specimens in a dose- and time- dependent manner (Figure 3). This indicates anaemia or iron deficiency, obstructive liver disease or liver dysfunction and signs of oxidative stress in NDEA intoxicated rats. Abnormal cell types were not detected in Group-1 (Control) specimen.

### Assessment of hepatic damage

**Figure 4:**
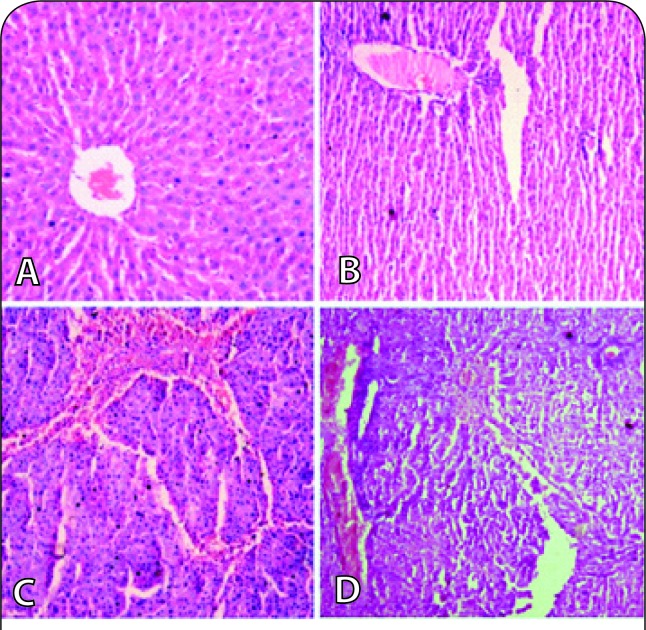
Haematoxylin & Eosin (H&E) stained liver biopsies of rats. (A) Group-1 (Control): showing normal lobular architecture, (B) Group-2 (Day-7, single dose of 200 mg/kg b.wt.): showing disruption in hepatic architecture with haemmorhage and neutrophilic infltration, (C) Group-3 (Day-7, single dose of 100 mg/kg b.wt.): showing damage in liver with severe haemmorhage and, (D) Group-4 (Day-14, 100 mg/kg b.wt./wk): showing dysplastic hepatocytes, haemmorhage, neutrophilic infiltration and damaged lobular architecture.

In order to visualise the hepatic damage caused by NDEA administration, haematoxylin and eosin (H&E) stained liver biopsies were examined and photographed (Figure 4). Group-1 (Control) specimen showed typical lobular architecture of the liver with intact parenchyma and normal shape and size of cell types. The stained liver sections of NDEA treated Group-2 animals exhibited disruption in cellular and lobular architecture with intense haemorrhage. Group-3 and -4 also displayed altered liver architecture, dysplastic hepatocytes, inflammation, neutrophilic infiltrations and severe haemorrhage in dose- and time-dependent manner.

### Molecular weight estimation and polypeptide profiling

**Figure 5:**
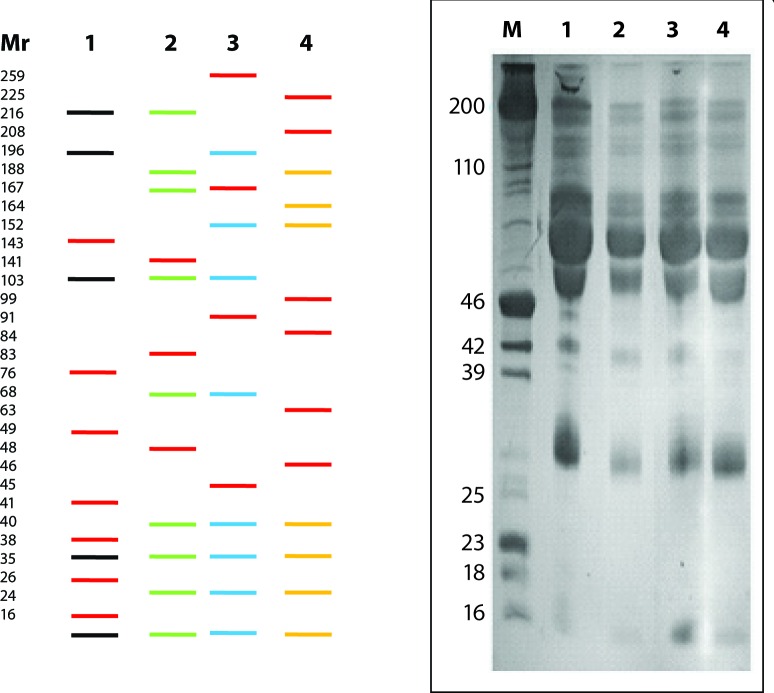
SDS-Polyacrylamide Gel Electrophoresis (SDS-PAGE) profiles of sera in control and NDEA treated rats. Left Panel shows only the polypeptide differences based on GelPro and Scion Imaging software analyses (Bands in red are novel whereas black, green, blue and yellow represent Group-1 (Control), NDEA-treated Group-2, -3 and -4. Right Panel depicts the comprehensive PAGE profiles of sera on 10% SDS-PAGE. M = Molecular weight marker; Lanes: 1–4 represents Group-1-4.

**Figure 6:**
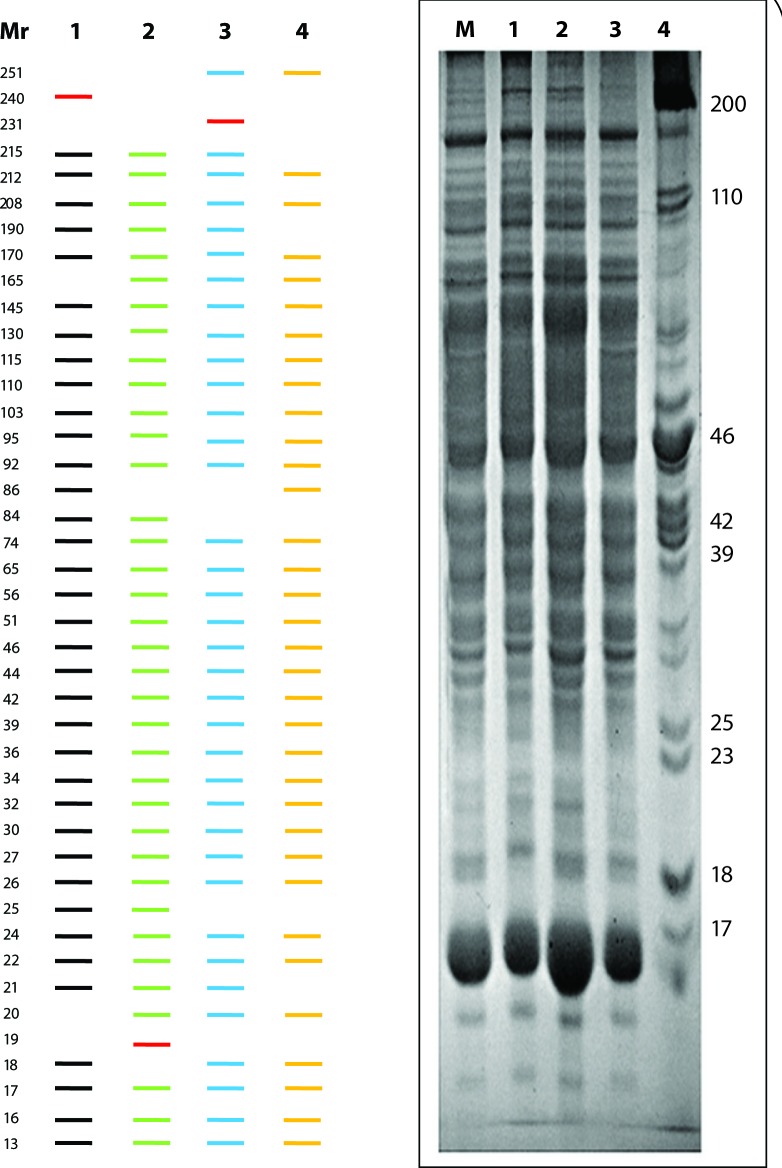
SDS-Polyacrylamide Gel Electrophoresis (SDS-PAGE) profiles of liver in control and NDEA treated rats. Left Panel shows only the polypetide diff erences based on GelPro and Scion Imaging software analyses (bands in red are novel whereas black, green, blue and yellow represent Group-1 (Control), NDEA-treated Group-2, -3 and -4. Right Panel demonstrates the comprehensive PAGE profiles of liver on 10% SDS-PAGE. M = Molecular weight marker; Lanes: 1–4 represents Group-1–4.

Representative SDS-PAGE profiles of serum and liver of control and NDEA treated rats are given in Figures 5 and 6. Software analysis using GelPro and Scion Image programmes revealed the presence of 30±5 and 42±4 polypeptides, irrespective of their electrophoretic mobilities, in serum and liver of rats, respectively. Treatment with NDEA caused quantitative as well as qualitative alterations in polypeptides in both tissues investigated. In the sera, seven polypeptides with molecular weights (M_r_) ~143, ~76, ~49, ~41, ~38, ~26 and ~23 kD were unique to Group-1 (Control) animals. Polypeptides with M_r_ ~141, ~83, ~48; ~259, ~167, ~91, ~45; and ~225, ~208, ~99, ~84, ~63, ~46 kD were found in sera of NDEA treated Group-2, -3 and -4, respectively. The polypeptide with M_r_ ~152 kD was detected in Group-3 and -4 only, while the polypeptide with M_r_ ~35 kD was uniformly present in the sera of all categories (Figure 5). In the liver, polypeptide with M_r_ ~240 kD was observed in Group-1 (Control) only. Polypeptides with M_r_ ~19 in Group-2 and ~231 kD in Group-3 were unique in their presence in the liver along with few other polypeptides with common existence in the NDEA treated categories (Figure 6). Polypeptides with M_r_ ~212, ~208, ~170, ~145, ~130, ~115, ~110, ~103, ~95, ~92, ~74, ~65, ~56, ~51, ~46, ~44, ~42, ~39, ~36, ~34, ~32, ~30, ~27, ~26, ~24, ~22, ~17, ~16 and ~13 kD were ubiquitous in the liver of all groups investigated, besides there were obvious quantitative differences. The variation in the overall polypeptide composition in the sera and liver of Group-1 and NDEA treated (Group-2 to 4) animals followed a dose-dependent profile.

## Discussion

*N*'-Nitrosodiethylamine (NDEA) is a potent hepatotoxin and hepatocarcinogen. It has been used in several experimental studies as an initiator agent to induce liver cancer. Since the liver is the main site of drug metabolism and detoxification of xenobiotics, study on the dose-dependent effect of NDEA on the liver of rats could be of interest to underline the causes of damage induced by NDEA *in vivo*. The biochemical data obtained during the present investigations showed that NDEA caused alterations in some serum enzymes and liver tissues. As a result of injury to liver cells by NDEA, several types of liver specific enzymes such as ALT, AST and ALP spill to the serum and become elevated. Various reports suggest that these enzymes are sensitive parameters to assess hepatic damage since they are cytoplasmic in location and are released into the circulation subsequent to cellular damage (Ahmad & Ahmad, 2012b; Rezai *et al*., 2013).

In some studies, sub-lethal doses of NDEA (LD5_0_= 280 mg/kg body weight) were used for various timings to generate hepatotoxicity or hepatocellular carcinoma (Brambilla *et al*., 1987; Zhang *et al*., 2012; Linza *et al*., 2013). In the present study, we selected three sub-lethal doses of NDEA and administered them to rats for 7–14 days to induce liver damage. The maximum damage was observed in the animals belonging to Group-4, as apparent from the weight loss of these animals. The degree of hepatic damage and the animal body/liver weight has been correlated in various studies (George & Chandrakasan, 2000; Ahmad *et al*., 2014a). Liver function test biomarkers, indicative for assessing initial liver dysfunctioning, also support the above findings showing the maximum change in Group-4 animals. These findings indicate that a repeated dose of NDEA of 100 mg/kg body weight/wk for two weeks appears to be more detrimental than a single dose of 200 mg/kg body weight/wk.

This study revealed changes in RBC rheology in NDEA treated rats (Figure 3) and showed altered cellular phenotypes, indicating iron deficiency or other liver dysfunction in exposed animals. Oxidation of NDEA leads to free-radical generation and haemolysis resulting in liberation of iron (Niki *et al*., 1988). Iron acts as pro-oxidant and favours free radical generation (Everse & Hsia, 1997; Armutcu *et al*., 2005). Since the RBC membrane is rich in polyunsaturated fatty acids, it is presumed that they are quite prone to free-radical mediated peroxidation and likely to cause cell membrane disruption (Armutcu *et al*., 2005; Ahmad *et al*., 2011). *N*'-Nitrosodiethylamine has ben reported to induce free radical generation that contributes to oxidative stress and cell injury through its metabolites and products (such as ethyl radicals). Hence, it is likely that polyunsaturated fatty acids present in the RBC membrane are degraded by these free radicals, resulting in altered RBC shapes.

Histochemical localisation by H & E staining of the control and NDEA treated rats displayed noticeable differences in liver architecture (Figure 4). Liver biopsies of the control group showed typical lobular architecture with intact parenchyma, central vein and sinusoidal spaces. Treatment of rats with NDEA damaged the liver architecture in a dose-dependent manner. In NDEA-treated liver specimens severe inflammation, haemorrhage, dysplastic hepatocytes and neutrophilic infiltrations were the characteristic features. Our histopathological data are in favour of the biochemical findings and clearly suggest that NDEA, like other nitrosamines (NDMA), may cause serious damage to the liver and interfere with its proper functioning (Ahmad *et al*., 2012; 2014a; Ali *et al*., 2014).

Polypeptide profiling was also carried out to screen sera and liver samples of Control and NDEA-treated animals. Our findings showed that at least three possible conditions of polypeptides occurred in the samples investigated: (1), Polypeptides present in all categories, *i.e.* ubiquitous or house-keeping (~35 kD in serum; ~212, ~208, ~170, ~145, ~130, ~115, ~110, ~103, ~95, ~92, ~74, ~65, ~56, ~51, ~46, ~44, ~42, ~39, ~36, ~34, ~32, ~30, ~27, ~26, ~24, ~22, ~17, ~16 and ~13 kD in liver); (2) Present in the control group (~143, ~76, ~49, ~41, ~38, ~26, ~23 kD in serum; ~240 kD in liver), or NDEA treated categories depending on the dose (~141, ~83, ~48 kD in Group-2; ~259, ~167, ~91, ~45 kD in Group-3; ~225, ~208, ~99, ~84, ~63, ~46 kD in Group-4 in serum; ~19 kD in Group-2; ~231 kD in Group-3; ~56 and ~30 kD in Group -4 in liver) and time (novel expression) and, (3) Present in all groups but displaying quantitative variations (differential expression). Overall 30±5 and 42±4 polypeptides, irrespective of their electrophoretic mobility, were detected through software analysis in serum and liver of rats, respectively. Differences in polypeptide composition may indicate a virtually different functional state of the tissue/organ as a consequence of NDEA treatment, as reported in several other toxicities (George & Chandrakasan, 2000; Ahmad *et al*., 2011; 2014b). Further investigations utilising 2D-Gel electrophoresis (2D-GE) are required to verify the differences in polypeptide composition of control and NDEA treated rats to propose candidate biomarkers. However, incongruity at any level in the number of polypeptides may be attributed to the sensitivity of the protocol and methodology used.

In summary, NDEA in the selected doses caused severe hepatic injury along with alterations in RBC rheology and polypeptide composition in rats, indicating disturbed biochemical homeostasis. However, maximum damage due to NDEA treatment was apparent in Group-4 receiving NDEA in 100 mg/kg body weight/wk for two weeks. It is noticeable that the difference in the level of damage produced in Group-2 and -4 animals may be due to the exposure time and rather than the amount of NDEA administered. Therefore, it is inferred that the hepatic damage and perturbed cellular functions may be the consequence of prolonged elevated oxidative stress, which is probably mediated by NDEA *via* the declining antioxidant milieu in Group-2 animals.
